# Changes in influenza vaccination coverage associated during the COVID‐19 pandemic in Japan

**DOI:** 10.1002/jgf2.462

**Published:** 2021-05-21

**Authors:** Dan Watanabe, Taku Harada, Juichi Hiroshige

**Affiliations:** ^1^ Department of General Medicine Showa University Hospital Tokyo Japan; ^2^ Department of Diagnostic and Generalist Medicine Dokkyo Medical University Hospital Tochigi Japan

**Keywords:** barriers to the vaccination, COVID‐19, influenza vaccine

## Abstract

Changes in influenza vaccination coverage during the COVID‐19 pandemic in Japan were limited. Not only changes in individual behavior and the threat of disease, but also vaccine dissemination policy based on system‐based interventions, including behavioral economic approaches, is desirable.

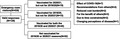

## CONFLICT OF INTEREST

The other authors have stated explicitly that there are no conflicts of interest in connection with this article.

## ETHICAL APPROVAL

This cross‐sectional study was approved by the ethical review board of the Showa University Koto Toyosu Hospital (No. 20T7050).

To the Editor,

Vaccination reduces the influenza rate by approximately 60% among individuals ≥16 years of age.^1^ In Japan, however, the influenza vaccination rate is only approximately 40% and has plateaued in recent years.^2^ According to the 4C model, barriers to vaccination include complacency, convenience, confidence, and calculation. Lack of confidence influenza vaccines, because of misconceptions and negative attitudes, has been the most frequently reported reason for not being vaccinated.^3^ Within the current pandemic situation, we were also interested in investigating the effect of COVID‐19 on decisions to follow through with influenza vaccination. To this end, we investigated the annual influenza vaccination coverage in Japan following the onset of the coronavirus disease (COVID‐19) pandemic, for the period of 2019/2020 and 2020/2021, including reasons for not being vaccinated during this period.

**FIGURE 1:**
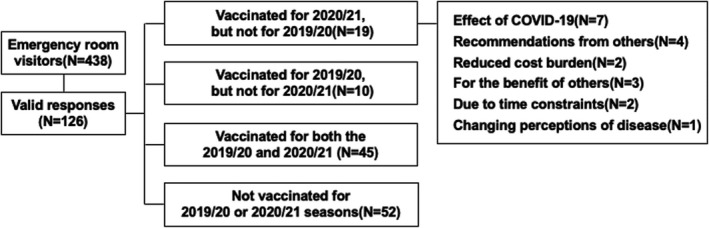
The number of people vaccinated with influenza vaccine in the 2019/20 and 2020/21 seasons and reasons for new vaccination in the 2020/21 season

This was a questionnaire‐based, cross‐sectional study of patients ≥16 years old who received treatment in our Department of General Medicine emergency room between January 1, 2021, and February 28, 2021. Patients were asked about their flu vaccination status and reasons for not being vaccinated. Excluded were individuals with an unknown vaccination history, those considering vaccination at a later date, and those who did not consent. Duplicate responses were eliminated. Among 438 questionnaires deployed, 126 (28.8%) valid responses were received. Respondents were female (44.4%), and the median age of respondents was 56.5 (range, 37.3–77.0) years. Overall, 10/126 (7.9%) were vaccinated in 2019/20 but not in 2020/21 and 19/126 (15.1%) were not vaccinated in 2019/2020 but were in 2020/2021. Additionally, 45/126 (35.7%) respondents were newly vaccinated in the 2020/21 season and 52 (41.3%) were not vaccinated in either period. Among newly vaccinated patients in 2020/2021, 7 (36.8%) cited COVID‐19 as the direct reason, with 4 (21.1%) citing a recommendation from others and two (10.5%) a reduced cost burden with reasons indirectly related to COVID‐19 (Figure 1). The overall influenza vaccination coverage was 43.7% (55/126 patients) in 2019/2020 and 50.8% (64/126 patients) in 2020/2021. Therefore, there was an increase in the rate of influenza vaccination during the COVID‐19 pandemic, although this increase was not significant (*p* = 0.137), with no change in this trend when only elderly individuals were considered (*n* = 52). We do note that this was a single‐center study and that there may be differences across regions. Other limitations of our study include the overall low response rate and the lack of evaluation of unvaccinated groups.

The World Health Organization recommends influenza vaccination to lower the pressure on medical institutions caused by the influenza within the context of the ongoing COVID‐19 pandemic.^4^ However, we did not identify a significant change in the influenza vaccination rate with the COVID‐19 epidemic. Even during the 2009/2010 H1N1 influenza pandemic, there was no significant change in the vaccination coverage.^2^ From a biomedical perspective, it seems that behavioral changes regarding seasonal influenza vaccination are challenging to achieve. A recent study indicated that an economic‐based intervention might improve vaccination compliance.^5^ Therefore, compliance with seasonal influenza vaccination might be positively influenced by policy and population‐based interventions, including behavioral economic approaches.
